# Supplementary material for: Antigenic cartography using sera from sequence-confirmed SARS-CoV-2 variants of concern infections reveals antigenic divergence of Omicron
Source: Immunity. 2022 Sep 13;55(9):1725–1731.e4. doi: 10.1016/j.immuni.2022.07.018 (PMC9353602; doi:10.1016/j.immuni.2022.07.018)
Supplement: Document S1. Figures S1 and Tables S2 and S3 [file mmc1.pdf]

Immunity, Volume *55*

Supplemental information

Antigenic cartography using sera from sequence-confirmed SARS-CoV-2 variants of concern infections reveals antigenic divergence of Omicron

Karlijn van der Straten, Denise Guerra, Marit J. van Gils, Ilja Bontjer, Tom G. Caniels, Hugo D.G. van Willigen, Elke Wynberg, Meliawati Poniman, Judith A. Burger, Joey H. Bouhuijs, Jacqueline van Rijswijk, Wouter Olijhoek, Marinus H. Liesdek, A.H. Ayesha Lavell, Brent Appelman, Jonne J. Sikkens, Marije K. Bomers, Alvin X. Han, Brooke E. Nichols, Maria Prins, Harry Vennema, Chantal Reusken, Menno D. de Jong, Godelieve J. de Bree, Colin A. Russell, Dirk Eggink, and Rogier W. Sanders

This PDF file includes:

Supplementary Figure 1 (Figure S1)
Supplementary Figure 2 (Figure S2)
Supplementary Table 2 (Table S2)
Supplementary Table 3 (Table S3)

Included as a separate excel file:

Supplementary Table 1 and corresponding legend (Table S1)

Supplementary Figure 1. Antigenic cartography reveals antigenic diversification of SARS-CoV-2, related to Figure 3A

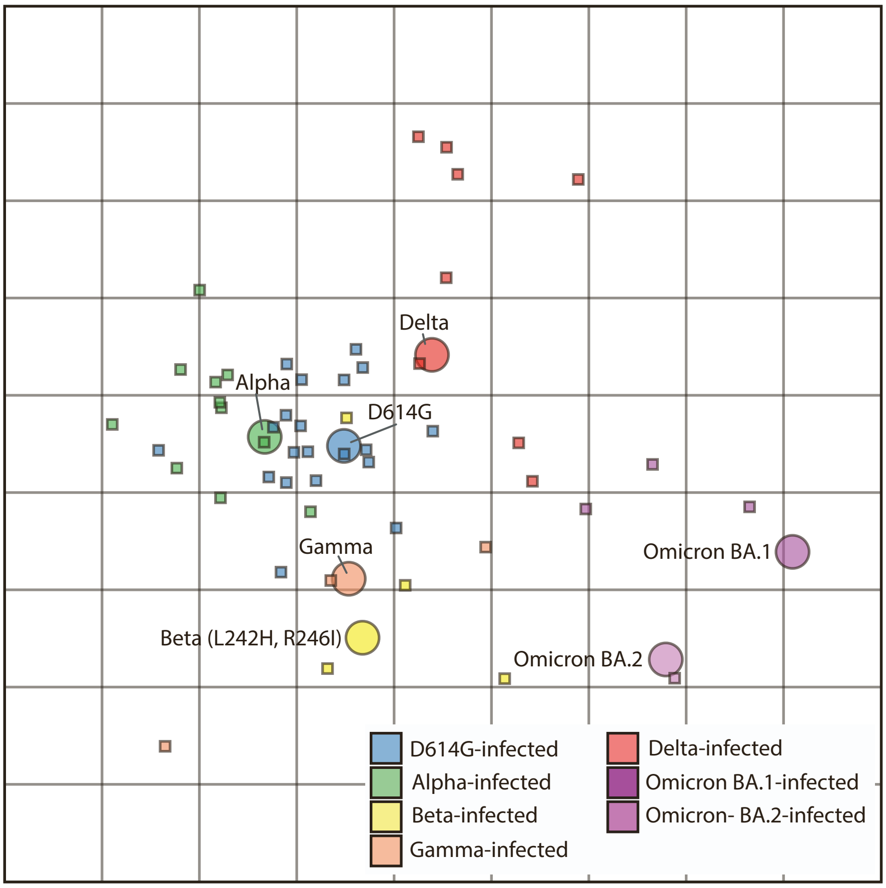

Antigenic map of SARS-CoV-2 VOCs based on convalescent SARS-CoV-2 infection sera. SARS-CoV-2 variants are shown as circles and sera are indicated as squares. Each square corresponds to sera of one individual and is coloured by the infecting SARS-CoV-2 variant. Both axes of the map are antigenic distance and each grid square (1 antigenic unit) represents a two-fold change in neutralization titre. The distance between points in the map can be interpreted as a measure of antigenic similarity, where the points more closely together show higher cross-neutralization and are therefore antigenically more similar. Compared to Figure 3A, this panel does not contain the Beta (Δ242-244) subvariant.

Supplementary Figure 2. Neutralization of convalescent VOC sera against two Beta subvariant pseudoviruses, related to Figure 1A

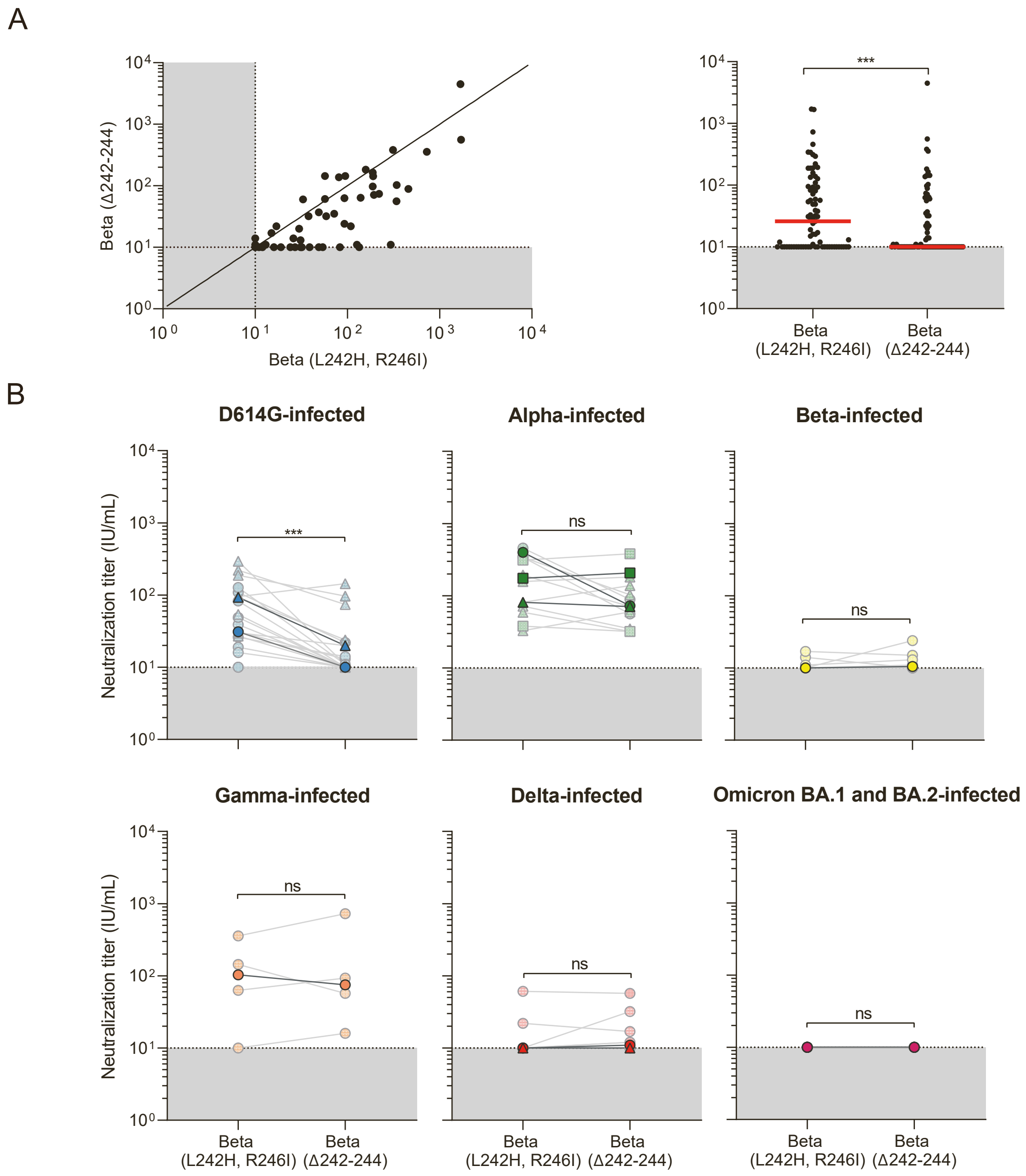

A. The left panel shows the correlation between the midpoint neutralization titres against both Beta subvariants used in this research, expressed in International Units per mL (IU/mL). In the right panel we studied the difference in neutralization titre against both Beta subvariants using a Wilcoxon signed rank test. Median neutralization titres are depicted as red bars. *** = $p<0.001$. B. Midpoint neutralization titres against both Beta subvariants in international Units per mL (IU/mL). The individuals are grouped per VOC they were infected with and plotted accordingly. Non-hospitalized patients are indicated with dots and hospitalized patients with triangles. The individuals that were infected with an Alpha variant that also included the E484K mutation are indicated in green squares. Median neutralization titres were compared using a Wilcoxon signed rank test. ns= non-significant, *** = $p< 0.001$.

Supplementary Table 2. Neutralization titres of convalescent SARS-CoV-2 sera against several VOCs, related to Figure 1-3.

Neutralization titres (ID50) were the serum dilution at which infectivity was inhibited 50% are converted to International Units per millilitre (IU/mL). Neutralization titers of post-vaccination sera can be found in Van Gils et al, Plos Medicine, 2022.

Infected strain	Participant ID	D614G	Alpha	Beta (L242H, R246I)	Beta (Δ242-244)	Gamma	Delta	Omicron BA.1	Omicron BA.2
D614G	COSCA-020	233	37	109	22	72	54	<2	81
D614G	COSCA-021	231	58	39	<10	35	29	<2	6
D614G	COSCA-022	28	11	<10	<10	<10	23	<2	<2
D614G	COSCA-023	34	13	19	<10	15	28	<2	3
D614G	COSCA-024	90	106	31	13	61	61	<2	7
D614G	COSCA-025	69	47	49	10	77	122	<2	9
D614G	COSCA-026	101	83	83	<10	38	97	<2	4
D614G	COSCA-028	189	119	26	14	81	217	21	55
D614G	COSCA-033	48	29	16	<10	28	36	<2	4
D614G	COSCA-034	34	27	<10	<10	<10	<10	<2	4
D614G	COSCA-112	929	1133	221	74	386	890	7	67
D614G	COSCA-113	122	97	31	<10	89	134	3	24
D614G	COSCA-114	237	249	127	11	131	68	3	15
D614G	COSCA-115	601	1095	93	24	386	384	112	13
D614G	COSCA-116	510	1005	188	97	482	230	9	157
D614G	COSCA-117	223	206	27	<10	92	48	5	7
D614G	COSCA-118	432	438	95	144	221	814	41	13
D614G	COSCA-119	430	220	30	20	186	326	11	19
D614G	COSCA-120	335	234	295	11	372	134	64	59
D614G	COSCA-123	852	463	54	<10	393	586	8	18
Alpha	AMCVIS1585	607	1366	194	71	436	344	23	13
Alpha	AMCVIS1871	215	683	342	103	414	557	20	<10
Alpha	AMCVIS2084	291	891	158	182	171	482	<10	11

Alpha	AMCVIS3484	124	872	33	60	93	232	13	<10
Alpha	AMCVIS5722	164	394	72	35	45	137	<10	<10
Alpha	AMCVIS9584	168	733	59	32	103	55	54	23
Alpha	COSCA-303	229	2103	341	56	122	108	40	16
Alpha	GGDVIS3343	1083	1658	458	89	1138	159	148	92
Alpha	VUVIS6992	263	788	81	138	149	252	4	<10
Alpha + E484K	COSCA-316	558	2091	312	382	730	181	24	36
Alpha + E484K	COSCA-320	59	213	38	32	82	16	9	6
Beta	COSCA-331	<10	<10	<10	14	<10	<10	<2	<2
Beta	COSCA-336	<10	16	15	17	34	<10	5	3
Beta	COSCA-337	<10	<10	<10	<10	<10	<10	2	5
Gamma	COSCA-309	57	51	57	144	87	<10	4	10
Gamma	COSCA-310	<10	28	93	63	224	<10	<2	4
Gamma	COSCA-324	<10	<10	16	<10	22	<10	<2	<2
Gamma	COSCA-334	537	326	726	357	761	403	798	725
Delta	COSCA-321	<10	14	<10	<10	<10	217	10	8
Delta	COSCA-322	11	10	<10	<10	<10	159	3	2
Delta	COSCA-323	50	54	12	<10	16	110	5	12
Delta	COSCA-325	105	37	<10	<10	<10	439	5	9
Delta	COSCA-327	<10	<10	<10	<10	<10	<10	<2	<2
Delta	COSCA-328	72	38	32	<10	21	150	70	30
Delta	COSCA-329	<10	<10	<10	<10	<10	19	<2	<2
Delta	COSCA-330	<10	<10	17	22	12	<10	<2	<2
Delta	COSCA-332	10	<10	<10	<10	10	59	<2	4
Delta	COSCA-333	114	78	57	61	121	1635	32	27
Delta	COSCA-335	<10	<10	12	<10	<10	27	8	7
Beta	COSCA-301	17	12	<10	<10	<10	11	<2	<2
Beta	COSCA-305	<10	<10	24	<10	67	<10	8	36

Beta	COSCA-306	<10	<10	11	<10	17	<10	<2	<2
Beta	COSCA-307	<10	<10	13	11	32	<10	2	2
Beta	COSCA-308	<10	<10	<10	<10	<10	<10	<2	<2
Omicron BA.1	COSCA-348	<10	<10	<10	<10	<10	<10	5	<2
Omicron BA.1	COSCA-349	<10	<10	<10	<10	<10	<10	24	15
Omicron BA.1	COSCA-350	<10	<10	<10	<10	<10	<10	61	21
Omicron BA.1	COSCA-351	<10	<10	<10	<10	<10	<10	22	2
Omicron BA.1 / BA.2	COSCA-352	<10	<10	<10	<10	<10	12	23	5
Omicron BA.1 / BA.2	COSCA-353	<10	<10	<10	<10	<10	11	90	22
Omicron BA.1	COSCA-354	<10	<10	<10	<10	<10	10	4	9
Omicron BA.2	COSCA-356	<10	16	<10	<10	<10	<10	<2	32
Omicron BA.2	COSCA-357	<10	<10	<10	<10	<10	<10	15	95
Omicron BA.2	COSCA-358	14	<10	<10	<10	<10	<10	16	95
Omicron BA.1	COSCA-360	<10	<10	<10	<10	<10	<10	3	37
Omicron BA.2	COSCA-361	<10	<10	<10	<10	<10	<10	<2	7

Supplementary Table 3. Exact p-values of statistical analysis of Figure 1A, Figure 2A and Figure S1.

Supplementary Table 3A. Statistical analysis of Figure 1A.

	**Infected individuals**						
	**Ancestral**	**Alpha**	**Beta**	**Gamma**	**Delta**	**Omicron BA.1**	**Omicron BA.2**
**Ancestral**		0,0010	0,6000	0,1750	0,0046	0,0729	0,25
**Alpha**	0,1208		0,6000	0,1750	0,0046	0,0729	0,25
**Beta (L242H, R246I)**	0,0002	0,0010		0,1750	0,0046	0,0729	0,25
**Beta (Δ242-244)**	0,0002	0,0010		0,3750	0,0059	0,0729	0,25
**Gamma**	0,0002	0,0010	0,3750		0,0046	0,0729	0,25
**Delta**	0,0183	0,0010	0,3750	0,1750		0,0729	0,25
**Omicron BA.1**	0,0001	0,0010	0,3750	0,3750	0,0046		0,25
**Omicron BA.2**	0,0001	0,0010	0,8750	0,1750	0,0046	0,2188	

ns	P > 0.05
*	P ≤ 0.05
**	P ≤ 0.01
***	P ≤ 0.001

Supplementary Table 3B. Statistical analysis of Figure 2A.

**P-values using Mann-Whitney U test**	**P-value**	
**Hospitalized patients**		
D614G vs Alpha infected	0,11	ns
**Non-hospitalized**		
D614G vs Beta(Δ242-244) infected	<0,0001	****
D614G vs Delta infected	0,46	ns
Beta (Δ242-244) vs Omicron BA.1 infected	0,088	ns

Supplementary Table 3C. Statistical analysis of Figure S1.

**P-values using Wilcoxon rank test**							
	Ancestral	Alpha	Beta	Gamma	Delta	Omicron BA.1	Omicron BA.2
Beta (L242H, R246I) vs Beta (Δ242-244)	0,0004	0,21	0,875	0,625	>0,99	>0,99	>0,99
