# Supplementary material for: Antigenic cartography using sera from sequence-confirmed SARS-CoV-2 variants of concern infections reveals antigenic divergence of Omicron
Source: Immunity. 2022 Sep 13;55(9):1725–1731.e4. doi: 10.1016/j.immuni.2022.07.018 (PMC9353602; doi:10.1016/j.immuni.2022.07.018)
Supplement: Document S2. Article plus supplemental information [file mmc3.pdf]

Article

Immunity

Antigenic cartography using sera from sequence-confirmed SARS-CoV-2 variants of concern infections reveals antigenic divergence of Omicron

Graphical abstract

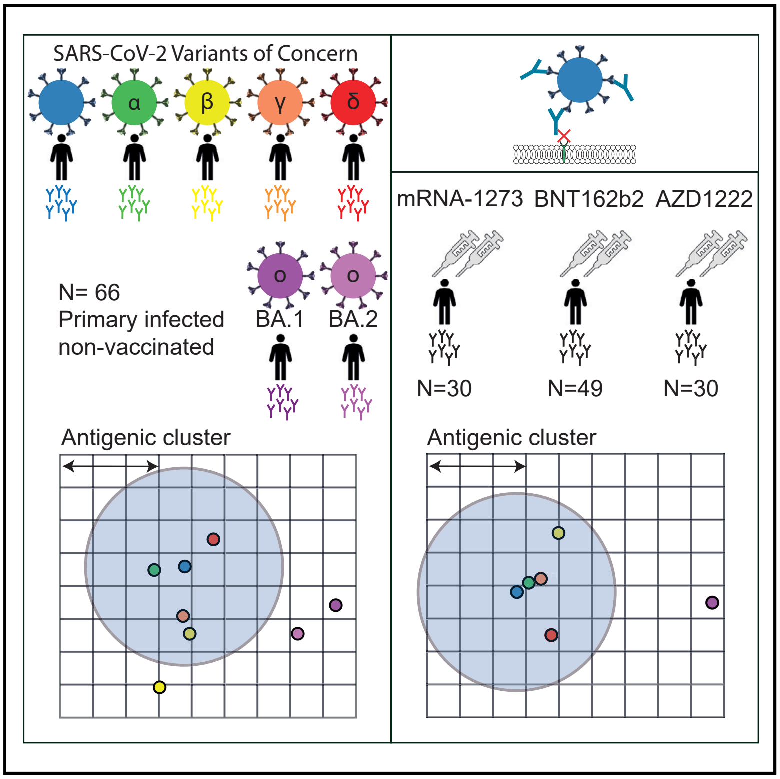

Authors

Karlijn van der Straten, Denise Guerra, Marit J. van Gils, ..., Colin A. Russell, Dirk Eggink, Rogier W. Sanders

Correspondence

c.a.russell@amsterdamumc.nl (C.A.R.),
dirk.eggink@rivm.nl (D.E.),
r.w.sanders@amsterdamumc.nl (R.W.S.)

In brief

Given the continued evolution of SARS-CoV-2, it is important to understand when and how to update vaccines to antigenically match circulating variants. van der Straten et al. demonstrate that infection with different SARS-CoV-2 variants leads to qualitatively different neutralizing antibody responses. Moreover, they show that Omicron represents a new cluster of antigenically distinct variants, which has implications for updating vaccines.

Highlights

- SARS-CoV-2 VOCs induce qualitatively different neutralizing antibody responses
- D614G and Alpha induce the strongest and broadest neutralizing antibody responses
- Omicron induces weaker neutralizing antibody responses
- Omicron BA.1 and BA.2 are antigenically distinct from the D614G strain

van der Straten et al., 2022, Immunity 55, 1725–1731
September 13, 2022 © 2022 The Authors. Published by Elsevier Inc.
https://doi.org/10.1016/j.immuni.2022.07.018

CellPress

Immunity

CellPress
OPEN ACCESS

Article

Antigenic cartography using sera from sequence-confirmed SARS-CoV-2 variants of concern infections reveals antigenic divergence of Omicron

Karlijn van der Straten,[1,2,3] Denise Guerra,[1,2] Marit J. van Gils,[1,2] Ilja Bontjer,[1,2] Tom G. Caniels,[1,2] Hugo D.G. van Willigen,[1,2] Elke Wynberg,[1,2,4] Meliawati Poniman,[1,2] Judith A. Burger,[1,2] Joey H. Bouhuijs,[1,2] Jacqueline van Rijswijk,[1,2] Wouter Olijhoek,[1,2] Marinus H. Liesdek,[1,2,3] A.H. Ayesha Lavell,[2,5] Brent Appelman,[6] Jonne J. Sikkens,[2,5] Marije K. Bomers,[2,5] Alvin X. Han,[1,2] Brooke E. Nichols,[1,2,7] Maria Prins,[3,4] Harry Vennema,[8] Chantal Reusken,[8] Menno D. de Jong,[1,2] Godelieve J. de Bree,[3] Colin A. Russell,[1,2,*] Dirk Eggink,[1,2,8,*] and Rogier W. Sanders[1,2,9,10,*]

[1]Amsterdam UMC Location University of Amsterdam, Department of Medical Microbiology and Infection Prevention, Laboratory of Experimental Virology, Meibergdreef 9, 1105 AZ Amsterdam, the Netherlands
[2]Amsterdam Institute for Infection and Immunity, Infectious Diseases, Amsterdam, the Netherlands
[3]Amsterdam UMC Location University of Amsterdam, Department of Internal Medicine, Meibergdreef 9, 1105 AZ Amsterdam, the Netherlands
[4]Department of Infectious Diseases, Public Health Service of Amsterdam, GGD, 1018 WT Amsterdam, the Netherlands
[5]Amsterdam UMC Location VU University Amsterdam, Department of Internal Medicine, Boelelaan 1117, 1081 HV Amsterdam, the Netherlands
[6]Amsterdam UMC Location University of Amsterdam, Center for Experimental and Molecular Medicine, Meibergdreef 9, 1105 AZ Amsterdam, the Netherlands
[7]Department of Global Health, Boston University School of Public Health, Boston, MA, USA
[8]Centre for Infectious Disease Control, National Institute for Public Health and the Environment, 3721 MA Bilthoven, the Netherlands
[9]Department of Microbiology and Immunology, Weill Medical College of Cornell University, New York, NY 10065, USA
[10]Lead contact
*Correspondence: c.a.russell@amsterdamumc.nl (C.A.R.), dirk.eggink@rivm.nl (D.E.), r.w.sanders@amsterdamumc.nl (R.W.S.)
https://doi.org/10.1016/j.immuni.2022.07.018

SUMMARY

Large-scale vaccination campaigns have prevented countless hospitalizations and deaths due to COVID-19. However, the emergence of SARS-CoV-2 variants that escape from immunity challenges the effectiveness of current vaccines. Given this continuing evolution, an important question is when and how to update SARS-CoV-2 vaccines to antigenically match circulating variants, similarly to seasonal influenza viruses where antigenic drift necessitates periodic vaccine updates. Here, we studied SARS-CoV-2 antigenic drift by assessing neutralizing activity against variants of concern (VOCs) in a set of sera from patients infected with viral sequence-confirmed VOCs. Infections with D614G or Alpha strains induced the broadest immunity, whereas individuals infected with other VOCs had more strain-specific responses. Omicron BA.1 and BA.2 were substantially resistant to neutralization by sera elicited by all other variants. Antigenic cartography revealed that Omicron BA.1 and BA.2 were antigenically most distinct from D614G, associated with immune escape, and possibly will require vaccine updates to ensure vaccine effectiveness.

INTRODUCTION

The coronavirus disease 2019 (COVID-19) pandemic, caused by the severe acute respiratory syndrome coronavirus 2 (SARS-CoV-2 virus), represents an enormous threat to human health and a burden to healthcare systems and economies worldwide. The unprecedented rapid development of efficacious vaccines fueled hope of curtailing this pandemic and permitting a return to a society without societal restrictions. However, genetic drift of SARS-CoV-2 resulted in the emergence of multiple variants of concern (VOCs) with a higher transmissibility compared with the ancestral strain, challenging the effectiveness of public health measures, vaccines, and/or therapeutics (World Health Organization, 2021). Based on this definition, the WHO designated the Alpha (Pango lineage B.1.1.7), Beta (B.1.351), Gamma (P.1), Delta (B.1.617.2), and Omicron (B.1.1.529, including sublineages BA.1 and BA.2) variants as VOCs. The Alpha, Beta, Gamma, and Delta VOCs have approximately 7–12 mutations in the spike protein (S), whereas Omicron BA.1 with 34 mutations, of which 3 deletions, and BA.2 with 28 mutations differ substantially from the ancestral strain (Figure 1A) (World Health Organization, 2021). Approximately half of Omicron's S mutations are located in the receptor binding domain (RBD) and eight mutations in the N-terminal domain (NTD), the two most

This is an open access article under the CC BY license (http://creativecommons.org/licenses/by/4.0/).

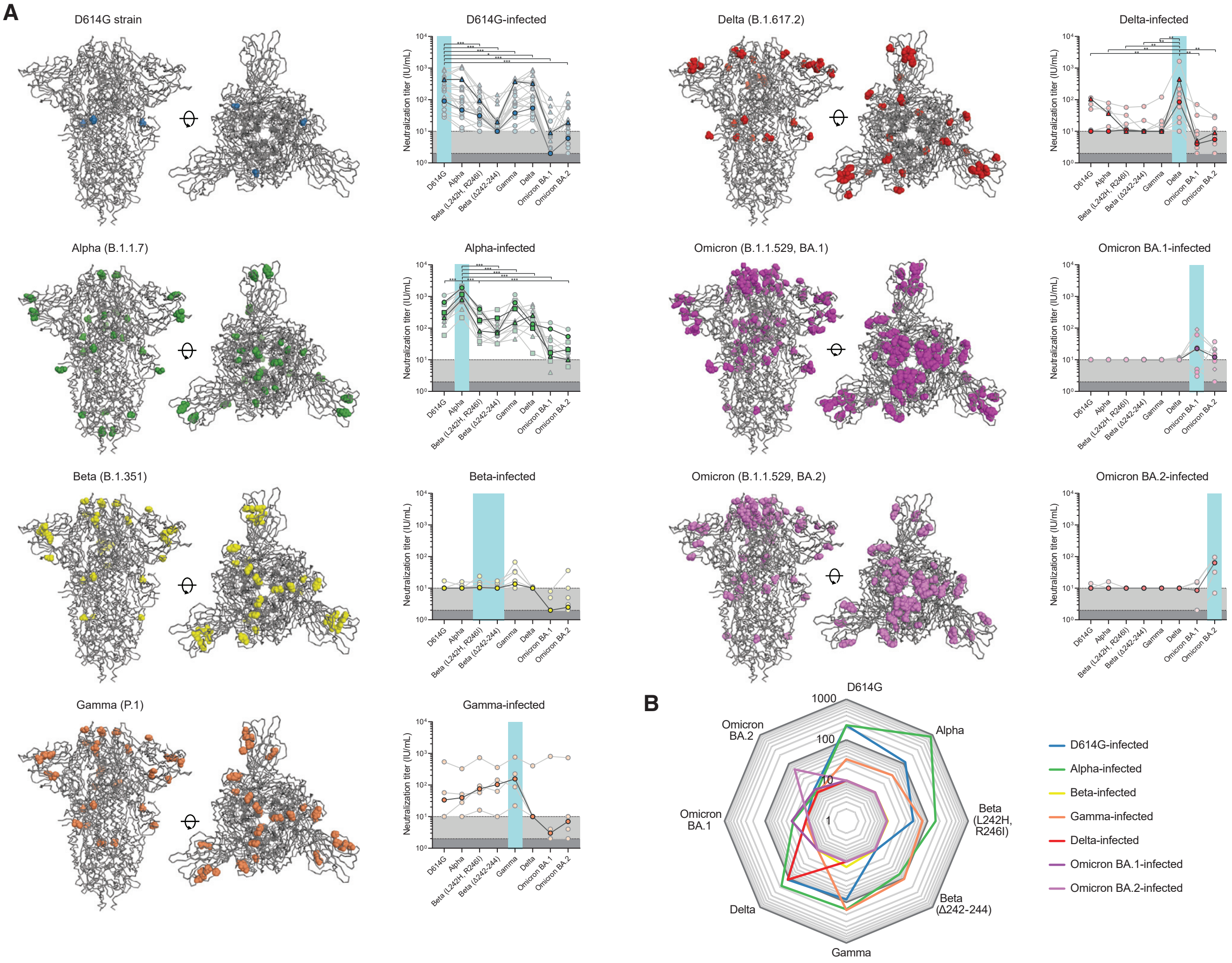

Figure 1. SARS-CoV-2 VOCs elicit diverse serum responses against homologous and heterologous strains

(A) Molecular models of SARS-CoV-2 S, highlighting the locations of mutations in the D614G strain (blue) and Alpha (green), Beta (yellow), Gamma (orange), Delta (red), Omicron BA.1 (magenta), and Omicron BA.2 (pink) variants. Midpoint neutralization titers against the VOCs in international units per mL (IU/mL). The individuals are grouped per VOC and plotted accordingly. Median neutralization titers are highlighted while the individual points are depicted with higher transparency. The light gray bar (10 IU/mL) indicates the neutralization cutoff for all strains except Omicron (cutoff 2 IU/mL, dark gray bar). Non-hospitalized patients are indicated with dots and hospitalized patients with triangles. The individuals who were infected with an Alpha strain that also included the E484K mutation are indicated in green squares. The two individuals in the Omicron BA.1 group who may have been infected with BA.2 instead of BA.1 are indicated in magenta diamonds (see also Table S1). The homologous neutralization is highlighted using a light blue bar. The Wilcoxon signed rank test with Benjamini-Hochberg correction was used to compare cross-neutralization titers with the homologous neutralization (see Table S3A for exact p values). Only statistically significant differences are indicated. *p < 0.005, **p < 0.01, *** p < 0.001, ****p < 0.0001.

(B) Spider plot of the median neutralization titer (IU/mL) of each group against all VOCs. A cutoff of 10 IU/mL is used for all strains.

important antigenic sites of S. Indeed, sera from COVID-19 patients infected with the ancestral strain and sera from vaccinees show up to 7- and 4-fold reductions in neutralization activity against Beta and Gamma, whereas 20- to 40-fold reductions are observed against Omicron BA.1 (Caniels et al., 2021; Garcia-Beltran et al., 2021; van Gils et al., 2022; Wilhelm et al., 2021).

However, the precise antigenic relationships among these VOCs are only starting to become clear. Understanding the differences between the serological antibody responses elicited by these variants is important to assess the risk of re-infections after natural infection and breakthrough infections after vaccination. For seasonal influenza viruses, this type of antigenic data is combined with virus genetic and epidemiological data to quantify the evolution of the virus and guide annual updates of the seasonal influenza virus vaccines. Antigenic cartography can be used to visualize antigenic relationships among viral variants (Fonville et al., 2014; Smith et al., 2004) and is routinely used in influenza virus vaccine strain selection. Until recently, antigenic cartography for SARS-CoV-2 has only been applied to cohorts of COVID-19 patients with uncertainty about their history of previous SARS-CoV-2 exposure and COVID-19 vaccinations, and without the usage of Omicron-infected human data (Liu et al.,

CellPress
OPEN ACCESS

Table 1. Sociodemographics and clinical characteristics

Variant of concern	N = (%)	Age (years) median (range)	Male	Hospital admission	Sequence confirmed	Time since symptom onset (days) median (range)
**D614G**	20 (30%)	53 (22–74)	9 (45%)	9 (45%)	0 (0%)	38 (30–45)
**Alpha**	11 (17%)	48 (25–76)	6 (55%)	9 (82%)	11 (100%)	39 (24–58)
Alpha + E484K	2/11	62 (48–76)	0	2/2	2/2	41 (24–58)
**Beta**	8 (12%)	41 (18–53)	3 (38%)	0	7 (88%)	39 (30–63)
**Gamma**	4 (6%)	35 (27–10)	2 (50%)	0	4 (100%)	40 (38–55)
**Delta**	11 (17%)	31 (19–63)	6 (55%)	1 (9%)	10 (91%)	46 (36–52)
**Omicron BA.1**	8 (12%)	31 (21–45)	4 (50%)	0	3 (38%)	44 (25–75)
**Omicron BA.2**	4 (6%)	51 (31–55)	4 (100%)	0	4 (100%)	40 (39–41)
Total	66	41 (18–76)	34 (52%)	21 (32%)	39 (59%)	40 (24–75)

A summary of the convalescent SARS-CoV-2 patients included in this study. See also Table S1 for a more comprehensive overview per individual.

2021; Mykytyn et al., 2022; Wilks et al., 2022). Here, we studied the (cross-)neutralizing antibody responses in sera from a well-defined population of convalescent individuals with a sequence-confirmed, or high likelihood of, primary infection by the D614G strain or Alpha, Beta, Gamma, Delta, or Omicron BA.1 or BA.2 variants and used this data as input for antigenic cartography to map the antigenic evolution of SARS-CoV-2.

RESULTS

Study population

We collected and analyzed a set of serum samples from 66 COVID-19 patients with a PCR-confirmed primary SARS-CoV-2 infection who did not receive any COVID-19 vaccinations. Blood was drawn 3–11 weeks after symptom onset (median 40 days, range 24–75 days), which corresponds with the peak of the antibody response (Tables 1 and S1) (Long et al., 2020). In total, n = 20 D614G-infected, n = 11 Alpha-infected, n = 8 Beta-infected, n = 4 Gamma-infected, n = 11 Delta-infected, n = 8 Omicron BA.1-infected, and n = 4 Omicron BA.2-infected participants were included. Of these participants, 39 had a sequence-confirmed VOC infection. The other 27 participants met our inclusion criteria of a high likelihood of VOC infection (see STAR Methods section; Table S1), of which 20 participants were assumed to be infected with the D614G strain as they were sampled before the emergence of any VOC in the Netherlands, but after D614G became dominant (Korber et al., 2020).

Magnitude and breadth of serum neutralization depend on the infecting SARS-CoV-2 VOC

We assessed the neutralizing capacity of the convalescent sera in a lentiviral-based pseudovirus neutralization assay against the D614G strain and the Alpha, two Beta, Gamma, Delta, and Omicron BA.1 and BA.2 variants (Figure 1A). The two Beta subvariants differ from each other in the NTD, where one Beta subvariant (L242H, R246I) is based on a very early available sequence while the other (Δ242–244) is more representative of the strains that circulated widely.

The highest neutralization titers were generally measured against the homologous virus, as might be expected (Figures 1A and 1B). Only the Beta-infected participants showed higher cross-neutralization titers against the Gamma variant compared with homologous neutralization, which is in line with other research (Wilks et al., 2022). This might be explained by the shared RBD mutations, as the RBDs of these variants only differ by one amino acid (K417N in Beta versus K417T in Gamma). Our analyses suffer somewhat from a disbalance in hospitalized versus non-hospitalized patients between the different VOC groups (Table 1). However, when comparing only non-hospitalized patients who generally have lower antibody levels compared with hospitalized patients, patients infected with the Alpha variant showed the strongest homologous neutralization (1881 IU/mL, range 1,658–2,103 IU/mL), followed by individuals infected with the Gamma variant (median of 156 IU/mL, range 22–761 IU/mL), D614G strain (median of 90 IU/mL, range 28–237 IU/mL), Delta variant (median of 85 IU/mL, range 10–1,635 IU/mL), and the Omicron BA.2 (median of 64 IU/mL, range 10–95 IU/mL) and Omicron BA.1 variants (median of 23 IU/mL, range 10–90 IU/mL) (Figure 2A). By contrast, none of the Beta-infected participants showed substantial homologous neutralization against either Beta subvariants.

Overall, the VOCs differed in their capacity to induce cross-neutralizing antibodies. Individuals infected with the Alpha variant induced the broadest response, followed by D614G strain-infected, Gamma-infected, and Delta-infected patients (Figure 2B), although there was substantial heterogeneity within all groups (Figures 1A and 2B). Notably, none of the patients infected with the Beta, Omicron BA.1, or Omicron BA.2 variants showed substantial cross-neutralization activity.

Reductions in neutralizing activity against the two Omicron variants were substantial in all groups (Figures 1A and 1B). Omicron neutralization dropped below the limit of detection (10 IU/mL or an ID_{50} of 100) in 44/66 of the studied individuals for BA.1 and 37/66 for BA.2. The median fold reduction of Omicron BA.1 neutralization versus homologous neutralization was 9-fold (range 1- to 93-fold) when considering all patients, 10-fold (range 3- to 93-fold) for patients infected with a D614G strain, 52-fold (range 11- to 89-fold) for Alpha-infected, 6-fold (range 1- to 22-fold) for Gamma-infected, and 6-fold (range 1- to 51-fold) for Delta-infected patients. The median fold reduction of Omicron BA.2 neutralization versus homologous neutralization was 5-fold (range −4- to 134-fold) when considering all patients, 8-fold (range 3- to 47-fold) for patients infected with a D614G strain, 68-fold (range 18- to 134-fold) for Alpha-infected,

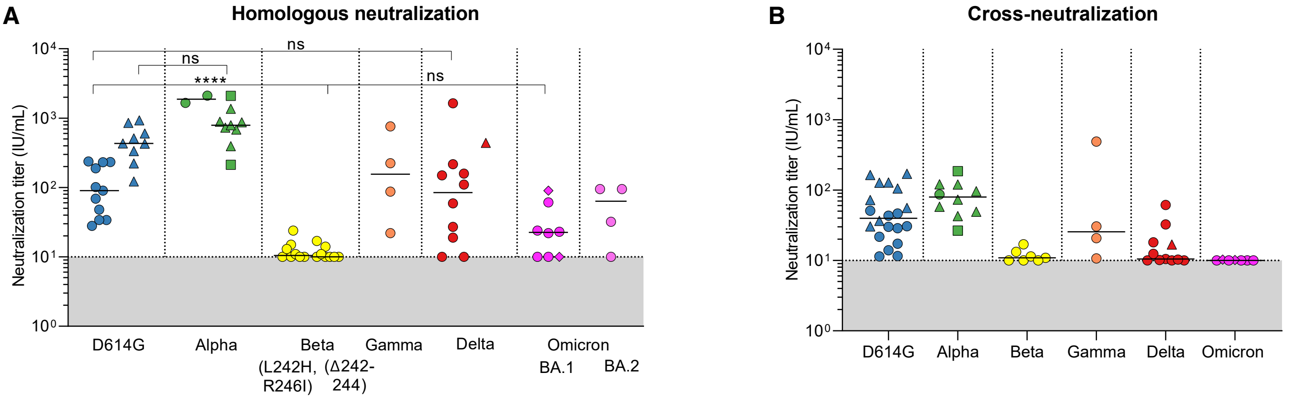

Figure 2. Alpha-infected and D614G-infected individuals show most potent and broad neutralizing response

(A) Midpoint neutralization titers against the VOCs in international units per mL (IU/mL). The individuals are grouped per VOC with which they were infected and plotted accordingly. Non-hospitalized patients are indicated with dots and hospitalized patients with triangles. The individuals who were infected with an Alpha variant that also included the E484K mutation are indicated in green squares. The two individuals in the Omicron BA.1 group who may have been infected with BA.2 instead of BA.1 are indicated in magenta diamonds (see also Table S1). A Mann-Whitney test is used to test for differences between group medians (black lines). ns, non-significant; **** $p < 0.0001$. See Table S3B for exact p values.

(B) Cross-neutralization is expressed as the geometric mean of the neutralization titers against all VOCs except the autologous strain in IU/mL. A cutoff of 10 IU/mL is used for all neutralization titers, as indicated by the gray bar.

6-fold (range 1- to 22-fold) for Gamma-infected, and 6-fold (range 1- to 60-fold) for Delta-infected patients.

Overall, we showed that exposure to different SARS-CoV-2 VOCs induces distinct serum responses, which differ in their abilities to neutralize homologous and heterologous virus strains.

Omicron BA.1 and BA.2 are antigenically distinct from the D614G strain and other VOCs

To explore the antigenic relationships among the VOCs, we used the neutralization data to construct a SARS-CoV-2 antigenic map (Figure 3A). In this map, homologous sera tend to cluster around the infecting strain, reflecting that homologous neutralization is dominant. The D614G and Alpha viruses cluster tightly together in the centre of the map, whereas the Beta (L242H, R246I), Gamma, and Delta variants all lie within 2 antigenic units (1 unit = 2-fold change in neutralization titer) of the D614G strain, suggesting a high degree of antigenic similarity. For influenza viruses, variants are considered to be antigenically similar in the case of antigenic distances below 3 antigenic units, i.e., an 8-fold change in neutralization titer, and different when above this threshold (Barr et al., 2014; Center for Disease Control and Prevention, 2021). By analogy, the D614G strain and Alpha, Beta (L242H, R246I), Gamma, and Delta variants belong to one antigenic cluster. Interestingly, the Beta (Δ242–244) subvariant is antigenically more distinct from the D614G strain, compared with Beta (L242H, R246I) (e.g., 3–4 units), implying that the deletion at region 242–244 has a substantial effect on antigenicity and illustrates the importance of the NTD as target of neutralizing antibodies and/or in modulating antigenicity of other domains by allosteric means. The distance between the main antigenic cluster and Omicron BA.1 and BA.2 variants is more than 4 antigenic units (>16-fold change in neutralization), implying that Omicron BA.1 and BA.2 are the antigenically most distinct SARS-CoV-2 variants (Figure 3A). One caveat is that it is unclear whether 2-fold changes in pseudovirus neutralization titers are directly comparable to 2-fold changes in hemagglutination inhibition assay titers used to define different antigenic clusters of influenza viruses. However, the change in neutralization between Omicron BA.1 and BA.2 and other variants of SARS-CoV-2, including the D614G strain, is striking.

We next used neutralization data from sera of 109 COVID-19 naive vaccinees receiving either two Moderna (mRNA-1273, n = 30), Pfizer/BioNTech (BNT162b2, n = 49), or AstraZeneca (AZD1222, n = 30) vaccines, which are all based on the ancestral S sequence to generate a second antigenic map (van Gils et al., 2022). This map (Figure 3B) agreed well with the convalescent sera map (Figure 3A) and corroborated that Omicron BA.1 represents a distinct antigenic variant from viruses currently included in vaccines. Interestingly, even though the distributions of sera from recipients of different vaccines overlap, there is a skew of sera of mRNA-1273 vaccinees toward Omicron BA.1, suggesting small differences in antigen stimulation among vaccine formulations considered here.

Taken together, these data indicate that whereas early variants belong to one antigenic cluster, the new Omicron BA.1 and BA.2 lineages are antigenically distinct from the D614G strain.

DISCUSSION

We have started to define the antigenic SARS-CoV-2 landscape after 2 years of antigenic drift, which should inform risk assessment of re-infections as well as strain selection for COVID-19 vaccine updates. We can draw several conclusions. First, homologous neutralization was usually stronger than heterologous neutralization. Second, heterologous responses were broadest and most potent in individuals infected with Alpha and D614G strains, whereas infection with Delta resulted in narrow-specificity responses. In addition, the individuals infected with the Beta and Omicron BA.1 variants, and to a lesser extent Omicron BA.2-infected individuals, developed weak neutralizing responses against any VOC, including the homologous strains,

CellPress
OPEN ACCESS

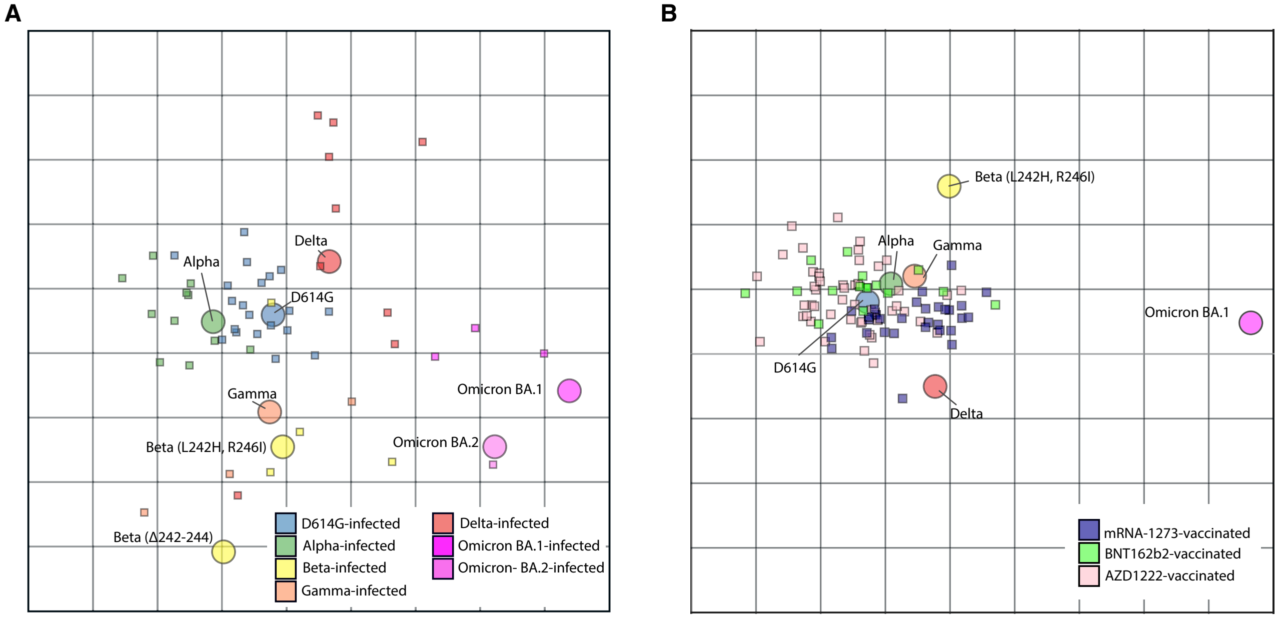

Figure 3. Antigenic cartography reveals antigenic diversification of SARS-CoV-2

(A) Antigenic map of SARS-CoV-2 VOCs based on convalescent SARS-CoV-2 infection sera. SARS-CoV-2 variants are shown as circles and sera are indicated as squares. Each square corresponds to sera of one individual and is colored by the infecting SARS-CoV-2 variant. Both axes of the map are antigenic distance, and each grid square (1 antigenic unit) represents a 2-fold change in neutralization titer. The distance between points in the map can be interpreted as a measure of antigenic similarity, where the points more closely together show higher cross-neutralization and are therefore antigenically more similar. Includes both Beta subvariants used in this study. Without the Beta (Δ242–244) subvariant. See Figure S1 for an antigenic map based on convalescent SARS-CoV-2 sera, including only one of the Beta subvariants (Beta L242H, R246I)

(B) Antigenic map of SARS-CoV-2 VOCs based on post-vaccination sera from individuals without prior SARS-CoV-2 infections. Each serum is colored by the vaccine that individual received.

suggesting that the S proteins of the Beta and both Omicron variants could be less immunogenic, compared with the S of other VOCs. The weak homologous neutralization and cross-neutralization of the Omicron BA.1 variant-infected individuals are in line with other studies (Mykytyn et al., 2022). Third, the D614G and Alpha strains are at the center of our antigenic map, which supports the use of the current COVID-19 vaccines based on the ancestral strain, in the case of the circulation of the Alpha, Beta (L242H, R246I), Gamma and Delta variants. Our data suggest that updated vaccines based on the Beta (L242H, R246I) or Delta variants would not have been appreciably more effective than the ancestral virus-based vaccines. However, the substantial reduction of neutralization in all groups against the Beta Δ242–244, but especially against the Omicron variants, indicates a high risk of re-infections and vaccine breakthrough cases when exposed to these VOCs (Eggink et al., 2022). The long antigenic distance between Omicron variants and the preceding variants in the antigenic map indicates that the current high rates of Omicron infections are at least partially associated with immune escape and that a vaccine update is required. While finishing this study, several other efforts to antigenically characterize VOCs became available (Mykytyn et al., 2022; Wilks et al., 2022). Our antigenic cartography is largely in accordance with these other studies.

As in the case of seasonal influenza viruses, the prospect of SARS-CoV-2 becoming an endemic virus with recurring outbreaks implies the need for surveillance of antigenic drift and possibly yearly administration of updated vaccines, especially for individuals at risk of severe COVID-19. Antigenic cartography efforts, such as those presented here, can inform future vaccine updates.

Limitations of the study

The weak homologous neutralization and cross-neutralization levels in individuals infected with the Beta variant are in contrast with the higher titers found by others (Cele et al., 2022; Liu et al., 2021; Rössler et al., 2022; Wilks et al., 2022). It is unlikely that this conflicting weak homologous neutralization is caused by a sequence mismatch between the strains causing the infection and the sequence used in our pseudoviruses, as both Beta subvariant pseudoviruses escape homologous neutralization of Beta sera, and the reduction of cross-neutralization of sera elicited by other VOCs against the Beta variants is in line with previous studies (Cele et al., 2022; Liu et al., 2021; Rössler et al., 2022; Wilks et al., 2022). One contributing factor for these discordant low (cross-)neutralization titers by the Beta variant includes cohort specific differences between studies, which is, however, hard to verify due to limited patient characteristic and demographic information available in publications.

Another limitation of this study includes the relatively small number of participants per group. For some VOCs, including the Beta and Gamma variants, the numbers of samples are limited due to the short period these variants circulated in the

Netherlands. For the groups with Omicron BA.1- and BA.2-infected individuals, the justification of the low numbers is more divergent. First, as the pandemic advances, many Omicron-infected individuals have experienced a previous SARS-CoV-2 infection. Second, the high vaccination rate in the Netherlands further limited the available eligible participants. In addition, non-vaccinated individuals are often hesitant to participate in scientific studies that might inform vaccine research. Although the low numbers are a limitation, the main conclusions are nevertheless clear and consistent with those of other studies (Cele et al., 2022; Liu et al., 2021; Rössler et al., 2022; Wilks et al., 2022), showing that Omicron BA.1 and BA.2 are antigenically most distinct from D614G, associated with immune escape, and possibly requiring vaccine updates to ensure vaccine effectiveness.

STAR★METHODS

Detailed methods are provided in the online version of this paper and include the following:

- KEY RESOURCES TABLE
- RESOURCE AVAILABILITY
  - Lead contact
  - Materials availability
  - Data and code availability
- EXPERIMENTAL MODEL AND SUBJECT DETAILS
  - Study population
  - Pseudovirus design
- METHOD DETAILS
  - SARS-CoV-2 pseudovirus neutralization assay
  - Antigenic cartography
- QUANTIFICATION AND STATISTICAL ANALYSIS

SUPPLEMENTAL INFORMATION

Supplemental information can be found online at https://doi.org/10.1016/j.immuni.2022.07.018.

ACKNOWLEDGMENTS

We thank all public health services (GGD) in the Netherlands for their help in contacting participants. We are also thankful to the study personnel and the participants of the COSCA, RECoVERED and the S3-study for their contribution to this research. Funding: R.W.S. and C.A.R. are recipients of Vici grants from the Netherlands Organization for Scientific Research (NWO no. 91818627 to R.W.S, no. 09150182010027 to C.A.R.). C.A.R. and A.X.H. are also supported by an ERC Consolidator Award no. 818353. This work was supported by the NWO ZonMw (grant agreement no. 10150062010002 to M.D.d.J. and no. 10430072110003 to G.J.d.B.) and the Public Health Service of Amsterdam Research & Development grant no. 21-14 to M. Prins (RECoVERED). J.J.S. and M.K.B. are recipients of the NWO grant agreement nos. 10430022010023 and 10430022010030, respectively.

AUTHOR CONTRIBUTIONS

Conceptualization, K.v.d.S., D.G., M.J.v.G., C.R., C.A.R., D.E., and R.W.S.; methodology, K.v.d.S., D.G., M.J.v.G., C.A.R., D.E., and R.W.S.; validation, K.v.d.S., D.G., M.J.v.G., and I.B.; formal analysis, K.v.d.S., A.X.H., B.E.N., and C.A.R.; investigation, I.B., M. Poniman, J.A.B., J.H.B., J.v.R., and W.O.; resources, K.v.d.S., I.B., H.D.G.v.W., E.W., M.H.L., A.H.A.L., B.A., J.J.S., M.K.B., M. Prins, H.V., C.R., M.D.d.J., and G.J.d.B.; writing – original draft, K.v.d.S., D.G., A.X.H., C.A.R., D.E., and R.W.S.; writing – review & editing, M.J.v.G., I.B., T.G.C., H.D.G.v.W., E.W., M. Poniman, J.A.B., J.H.B., J.v.R., W.O., M.H.L., A.H.A.L., B.A., J.J.S., M.K.B., B.E.N., M. Prins, H.V., C.R., M.D.d.J., and G.J.d.B.; visualization, K.v.d.S.,T.G.C., A.X.H., C.A.R., and R.W.S.; supervision, C.A.R., D.E., and R.W.S.; project administration, K.v.d.S. and D.G.; funding acquisition, J.J.S., M.K.B., A.X.H., M.Prins, M.D.d.J., G.J.d.B., C.A.R., and R.W.S.

DECLARATION OF INTERESTS

The authors declare no competing interests.

Received: January 7, 2022
Revised: May 26, 2022
Accepted: July 26, 2022
Published: August 5, 2022

REFERENCES

Barr, I.G., Russell, C., Besselaar, T.G., Cox, N.J., Daniels, R.S., Donis, R., Engelhardt, O.G., Grohmann, G., Itamura, S., Kelso, A., et al. (2014). WHO recommendations for the viruses used in the 2013–2014 Northern Hemisphere influenza vaccine: epidemiology, antigenic and genetic characteristics of influenza A(H1N1)pdm09, A(H3N2) and B influenza viruses collected from October 2012 to January 2013. Vaccine *32*, 4713–4725. https://doi.org/10.1016/j.vaccine.2014.02.014.

Caniels, T.G., Bontjer, I., van der Straten, K., Poniman, M., Burger, J.A., Appelman, B., Lavell, A.H.A., Oomen, M., Godeke, G.J., Valle, C., et al. (2021). Emerging SARS-CoV-2 variants of concern evade humoral immune responses from infection and vaccination. Sci. Adv. *7*, eabj5365. https://doi.org/10.1126/sciadv.abj5365.

Cele, S., Karim, F., Lustig, G., San, J.E., Hermanus, T., Tegally, H., Snyman, J., Moyo-Gwete, T., Wilkinson, E., Bernstein, M., et al. (2022). SARS-CoV-2 prolonged infection during advanced HIV disease evolves extensive immune escape. Cell Host Microbe *30*, 154–162.e5. https://doi.org/10.1016/j.chom.2022.01.005.

Center for Disease Control and Prevention (2021). Antigenic characterization. https://www.cdc.gov/flu/about/professionals/antigenic.htm.

CoVariants (2022). Overview of variants in countries. https://covariants.org/per-country.

Eggink, D., Andeweg, S.P., Vennema, H., van Maarseveen, N., Vermaas, K., Vlaemynck, B., Schepers, R., van Gageldonk-Lafeber, A.B., van den Hof, S., Reusken, C.B., and Knol, M.J. (2022). Increased risk of infection with SARS-CoV-2 Omicron BA.1 compared with Delta in vaccinated and previously infected individuals, the Netherlands, 22 November 2021 to 19 January 2022. Euro Surveill. *27*, 2101196. https://doi.org/10.2807/1560-7917.ES.2022.27.4.2101196.

Fonville, J.M., Wilks, S.H., James, S.L., Fox, A., Ventresca, M., Aban, M., Xue, L., Jones, T.C., Le, N.M.H., Pham, Q.T., et al. (2014). Antibody landscapes after influenza virus infection or vaccination. Science *346*, 996–1000. https://doi.org/10.1126/science.1256427.

Garcia-Beltran, W.F., St Denis, K.J., Hoelzemer, A., Lam, E.C., Nitido, A.D., Sheehan, M.L., Berrios, C., Ofoman, O., Chang, C.C., Hauser, B.M., et al. (2021). mRNA-based COVID-19 vaccine boosters induce neutralizing immunity against SARS-CoV-2 Omicron variant. Preprint at medRxiv. https://doi.org/10.1101/2021.12.14.21267755.

Grobben, M., van der Straten, K., Brouwer, P.J., Brinkkemper, M., Maisonnasse, P., Dereuddre-Bosquet, N., Appelman, B., Lavell, A.A., van Vught, L.A., Burger, J.A., et al. (2021). Cross-reactive antibodies after SARS-CoV-2 infection and vaccination. eLife *10*, e70330. https://doi.org/10.7554/eLife.70330.

Korber, B., Fischer, W.M., Gnanakaran, S., Yoon, H., Theiler, J., Abfalterer, W., Hengartner, N., Giorgi, E.E., Bhattacharya, T., Foley, B., et al. (2020). Tracking changes in SARS-CoV-2 spike: evidence that D614G increases infectivity of the COVID-19 virus. Cell *182*, 812–827.e19. https://doi.org/10.1016/j.cell.2020.06.043.

CellPress
OPEN ACCESS

Kristiansen, P.A., Page, M., Bernasconi, V., Mattiuzzo, G., Dull, P., Makar, K., Plotkin, S., and Knezevic, I. (2021). WHO International Standard for anti-SARS-CoV-2 immunoglobulin. Lancet *397*, 1347–1348. https://doi.org/10.1016/S0140-6736(21)00527-4.

Liu, C., Ginn, H.M., Dejnirattisai, W., Supasa, P., Wang, B., Tuekprakhon, A., Nutalai, R., Zhou, D., Mentzer, A.J., Zhao, Y., et al. (2021). Reduced neutralization of SARS-CoV-2 B.1.617 by vaccine and convalescent serum. Cell *184*, 4220–4236.e13. https://doi.org/10.1016/j.cell.2021.06.020.

Long, Q.X., Liu, B.Z., Deng, H.J., Wu, G.C., Deng, K., Chen, Y.K., Liao, P., Qiu, J.F., Lin, Y., Cai, X.F., et al. (2020). Antibody responses to SARS-CoV-2 in patients with COVID-19. Nat. Med. *26*, 845–848. https://doi.org/10.1038/s41591-020-0897-1.

Mykytyn, A.Z., Rissmann, M., Kok, A., Rosu, M.E., Schipper, D., Breugem, T.I., van den Doel, P.B., Chandler, F., Bestebroer, T., de Wit, M., et al. (2022). Antigenic cartography of SARS-CoV-2 reveals that Omicron BA.1 and BA.2 are antigenically distinct. Sci. Immunol. eabq4450. https://doi.org/10.1126/sciimmunol.abq4450.

Rijksinstituut voor Volksgezondheid en Milieu (RIVM) (2022). Resultaten kiemsurveillance. https://www.rivm.nl/sites/default/files/2022-04/Kiemsurveillance%2020220401%20tabel%20NL.pdf.

Rössler, A., Riepler, L., Bante, D., von Laer, D., and Kimpel, J. (2022). SARS-CoV-2 Omicron variant neutralization in serum from vaccinated and convalescent persons. N. Engl. J. Med. *386*, 698–700. https://doi.org/10.1056/NEJMc2119236.

Schmidt, F., Weisblum, Y., Muecksch, F., Hoffmann, H.H., Michailidis, E., Lorenzi, J.C.C., Mendoza, P., Rutkowska, M., Bednarski, E., Gaebler, C., et al. (2020). Measuring SARS-CoV-2 neutralizing antibody activity using pseudotyped and chimeric viruses. J. Exp. Med. *217*, e20201181. https://doi.org/10.1084/jem.20201181.

Smith, D.J., Lapedes, A.S., de Jong, J.C., Bestebroer, T.M., Rimmelzwaan, G.F., Osterhaus, A.D., and Fouchier, R.A. (2004). Mapping the antigenic and genetic evolution of influenza virus. Science *305*, 371–376. https://doi.org/10.1126/science.1097211.

van Gils, M.J., Lavell, A., van der Straten, K., Appelman, B., Bontjer, I., Poniman, M., Burger, J.A., Oomen, M., Bouhuijs, J.H., van Vught, L.A., et al. (2022). Antibody responses against SARS-CoV-2 variants induced by four different SARS-CoV-2 vaccines in health care workers in the Netherlands: a prospective cohort study. PLoS Med. *19*, e1003991. https://doi.org/10.1371/journal.pmed.1003991.

Wilhelm, A., Widera, M., Grikscheit, K., Toptan, T., Schenk, B., Pallas, C., Metzler, M., Kohmer, N., Hoehl, S., Helfritz, F.A., et al. (2021). Reduced neutralization of SARS-CoV-2 Omicron variant by vaccine sera and monoclonal antibodies. Preprint at medRxiv. https://doi.org/10.1101/2021.12.07.21267432.

Wilks, S.H., Mühlemann, B., Shen, X., Türeli, S., LeGresley, E.B., Netzl, A., Caniza, M.A., Chacaltana-Huarcaya, J.N., Corman, V.M., Daniell, X., et al. (2022). Mapping SARS-CoV-2 antigenic relationships and serological responses. Preprint at bioRxiv. https://doi.org/10.1101/2022.01.28.477987.

World Health Organization (2021). Tracking SARS-CoV-2 variants. https://www.who.int/en/activities/tracking-SARS-CoV-2-variants/.

Wynberg, E., van Willigen, H.D.G., Dijkstra, M., Boyd, A., Kootstra, N.A., van den Aardweg, J.G., van Gils, M.J., Matser, A., de Wit, M.R., Leenstra, T., et al. (2021). Evolution of COVID-19 symptoms during the first 12 months after illness onset. Clin. Infect. Dis. https://doi.org/10.1093/cid/ciab759.

STAR★METHODS

KEY RESOURCES TABLE

REAGENT or RESOURCE	SOURCE	IDENTIFIER
Media and buffers		
DMEM media	Gibco	Cat#: 11966025
Opti-MEM I reduced serum media	Gibco	Cat#: 15392402
Phosphate-buffered saline (PBS)	Gibco	Cat#: 15326239
Bacterial and virus strains		
Chemically competent DH5α *Escherichia coli*	Thermo Fisher Scientific	Cat#: 12879416
Biological samples		
Human sera, convalescent SARS-Cov-2	This study	N/A
Human sera, post-COVID-19 vaccination	(van Gils et al., 2022)	N/A
Chemicals, peptides, and recombinant proteins		
FastDigest SacI	Thermo Fisher Scientific	Cat#: 10324720
FastDigest ApaI	Thermo Fisher Scientific	Cat#: 10450280
Polyethylenimine hydrochloride (PEI) MAX	PolySciences	Cat#: 24765-1
Trypsin-EDTA	Gibco	Cat#: 25-200-056
GlutaMAX	Gibco	Cat#: 35050061
Glycylglycine, 99+%	Acros Organics/Thermo Scientific	Cat#: 120141000
Magnesium sulfate heptahydrate, 99.5%, for analysis, Thermo Scientific™	Acros Organics/ Thermo Scientific	Cat#: AC21311500
EGTA (ethylene glycol bis(2-aminoethyl ether)-N,N,N',N'-tetraacetic acid) ≥97%, Ultra Pure Grade	VWR	Cat#: 0732-100G
Triton™ X-100 (Electrophoresis), Fisher BioReagents™	Fisher BioReagents	Cat#: BP151-500
Poly-L-Lysine Hydrobromide	Sigma-Aldrich	Cat#: P1399
Critical commercial assays		
Gibson Assembly	New England BioLabs	N/A
QuikChange Site-Directed Mutagenesis Kit	Agilent Technologies	Cat#: 200523
Experimental models: Cell lines		
HEK293T cells	American Type Culture Collection	CRL-11268
HEK-293T-hACE2	(Schmidt et al., 2020)	RRID:CVCL_A7UK
Oligonucleotides		
SARS-CoV-2 D614G spike gene fragment	Integrated DNA Technologies	GenBank:MT449663.1 Mutations in this paper
SARS-CoV-2 Alpha spike gene fragment	Integrated DNA Technologies	GenBank:MT449663.1 Mutations in this paper
SARS-CoV-2 Beta spike gene fragment	Integrated DNA Technologies	GenBank:MT449663.1 Mutations in this paper
SARS-CoV-2 Gamma spike gene fragment	Integrated DNA Technologies	GenBank:MT449663.1 Mutations in this paper
SARS-CoV-2 Delta spike gene fragment	Integrated DNA Technologies	GenBank:MT449663.1 Mutations in this paper
SARS-CoV-2 Omicron BA.1 spike gene fragment	Integrated DNA Technologies	GenBank:MT449663.1 Mutations in this paper
SARS-CoV-2 Omicron BA.2 spike gene fragments	Integrated DNA Technologies	GenBank:MT449663.1 Mutations in this paper
Recombinant DNA		
pCR3 SARS-CoV-2–$S_{\Delta 19}$ expression plasmid	GenBank	ID: MT449663.1
pHIV-1_{NL43} ΔEnv-NanoLuc reporter virus plasmid	(Schmidt et al., 2020)	N/A

(*Continued on next page*)

Continued

REAGENT or RESOURCE	SOURCE	IDENTIFIER
Software and algorithms		
ACMACS antigenic cartography software	https://acmacs-web.antigenic-cartography.org/	N/A
GraphPad Prism 8.3.0	GraphPad	RRID:SCR_002798; http://www.graphpad.com/
Microsoft Excel	Microsoft	RRID:SCR_016137; https://www.microsoft.com/en-gb/
Other		
Nano-Glo Luciferase Assay System	Promega	Cat#: N1130
GloMax system	Turner BioSystems	Cat#: 9101-002

RESOURCE AVAILABILITY

Lead contact

Further information and requests for resources and reagents should be directed to and will be fulfilled by the lead contact, Rogier W. Sanders (r.w.sanders@amsterdamumc.nl).

Materials availability

This study did not generate new unique reagents.

Data and code availability

- Patient characteristics and neutralization data have been deposited as supplemental information associated with this manuscript (Tables S1 and S2, respectively) and are publicly available as of the date of publication. Other references to data used in the study are listed in the key resources table. Additional supplemental information are available from Mendeley Data at https://doi.org/10.17632/58mjpm9mvb.1.
- This paper does not report original code.
- Any additional information required to reanalyse the data reported in this paper is available from the lead contact upon request.

EXPERIMENTAL MODEL AND SUBJECT DETAILS

Study population

66 adults (aged 18 to 76) with a PCR proven primary SARS-CoV-2 infection were included in the COSCA-study (NL 73281.018.20) or the RECOVERED study (NL73759.018.20) between June 2020 and April 2022 at Amsterdam UMC and via the Dutch national SARS-CoV-2 sequence surveillance program as described previously (Grobben et al., 2021; Wynberg et al., 2021). In short, 3-11 weeks after symptom onset, blood, patient demographics, time between symptom onset and sampling, and admission status were collected (Tables 1 and S1). The diagnostic oropharyngeal swab was available for 39 participants and were used to determine the SARS-CoV-2 strain causing the infection. The remaining 27 SARS-CoV-2 infected participants fell within the following inclusion criteria: (1) $\geq$95% of circulating strains at time of symptom onset belonged the suspected VOC of infection or (2) $\geq$75% of circulating strains at the time of symptom onset belonged the suspected VOC of infection AND a household member had a concurrent sequence confirmed infection with that particular VOC. Prevalence data of CoVariants.org and the National Institute for Public Health and the Environment were used to determine the current prevalence of a VOC (CoVariants, 2022; Rijksinstituut voor Volksgezondheid en Milieu [RIVM]). Most individuals of which no sequence confirmation of the infected strain was available, were presumed to be infected with de D614G variant (n=20) as they were sampled before the emergence of any VOC in the Netherlands and after D614G became predominant in the Netherlands (Korber et al., 2020). More details about the remaining n=7 individuals can be found in the Table S1. The two Omicron individuals that may have been infected by either BA.1 or BA.2 are indicated as diamonds in all graphs. Two of the individuals infected with an Alpha strain harbouring the E484K mutation are indicated as squares in all graphs.

Neutralization data on COVID-19 naive vaccinee sera were kindly provided by the S3-study of the Amsterdam UMC, The Netherlands(NL73478.029.20) (van Gils et al., 2022). In short, post-vaccination sera was obtained approximately four weeks after the second doses of either Moderna (mRNA-1273), Pfizer/BioNTech (BNT162b2), or AstraZeneca (AZD1222). Post-vaccination serum after Janssen (Ad26.COV2.S) were excluded from analysis because they did not have enough non-threshold titres to be included in the map.

All above mentioned studies were conducted at the Amsterdam University Medical Centres, the Netherlands, and approved by the local ethical committees. All individuals provided written informed consent before participating.

Pseudovirus design

The D614G strain and the Alpha pseudovirus constructs contained the following mutations: D614G in D614G strain; deletion (Δ) of H69, V70 and Y144, N501Y, A570D, D614G, P681H, T716I, S982A, and D1118H in Alpha. The two Beta subvariants differ from each other in the NTD region 242-246, where one Beta subvariant (L242H, R246I) is based on a very early available sequence while the other (Δ242-244) is retrospectively more representative for the predominant circulating strains. These two Beta pseudovirus constructs contain therefore the following mutations: L18F, D80A, D215G, L242H, R246I, K417N, E484K, N501Y, D614G, and A701V in Beta (L242H, R246I); L18F, D80A, D215G, Δ242-244, K417N, E484K, N501Y, D614G, and A701V in Beta (Δ242-244). Only the D614G infected individuals showed statistically significant reduced neutralization against the Beta (Δ242-244) subvariant compared to the Beta (L242H, R246I) subvariant (Figure S2, Table S3C for exact P-values). The Gamma pseudovirus constructs contained the following mutations: L18F, T20N, P26S, D138Y, R190S, K417T, E484K, N501Y, D614G, H655Y, and T1027I in Gamma; This Gamma pseudovirus construct differs from the predominant strain in that it lacks a V1176F back bone mutation. However, it is not likely that this mutation, positioned at the S2 domain of the S, will affect escape of neutralization substantially. The Delta and Omicron BA.1 and BA.2 pseudovirus constructs contained the following mutations: T19R, G142D, E156G, Δ157-158, L452R, T478K, D614G, P681R and D950N in Delta; A67V, Δ69-70, T95I, G142D, Δ143-145, Δ211, L212I, ins214EPE, G339D, S371L, S373P, S375F, K417N, N440K, G446S, S477N, T478K, E484A, Q493K, G496S, Q498R, N501Y, Y505H, T547K, D614G, H655Y, N679K, P681H, N764K, D796Y, N856K, Q954H, N969K, L981F in Omicron BA.1; and T19I, L24S, Δ125/127, G142D, V213G, G339D, S371F, S373P, S375F, T376A, D405N, R408S, K417N, N440K, S477N, T478K, E484A, Q493R, Q498R, N501Y, Y505H, D614G, H655Y, N679K, P681H, N764K, D796Y, Q954H, N969K in Omicron BA.2. The Omicron BA.1 strain used here harbors a Q493K mutation, while the predominant Omicron BA.1 harbors a Q493R mutation. This mutation did not impacted neutralization of several monoclonal SARS-CoV-2 antibodies tested (data not shown). The spike constructs were ordered as gBlock gene fragments (Integrated DNA Technologies) and cloned SacI and ApaI in the pCR3 SARS-CoV-2–S_{Δ}19 expression plasmid (GenBank: MT449663.1) using Gibson Assembly (Thermo Fisher Scientific). The pseudovirus constructs were made using the QuikChange Site-Directed Mutagenesis Kit (Agilent Technologies) and verified by using Sanger sequencing. Pseudoviruses were procedures by cotransfecting HEK293T cells (American Type Culture Collection, CRL-11268) with the pCR3 SARS-CoV-2-S_{Δ}19 expression plasmid and the pHIV-1_{NL43} ΔEnv-NanoLuc reporter virus plasmid. Transfection takes place in cell culture medium (DMEM), supplemented with 10% fetal bovine serum, penicillin (100 U/ml), streptomycin (100 g/ml). Medium is refreshed once 6-8 hours after transfection. 48 hours after the transfection, cell supernatants containing the pseudovirus were harvested and stored at -80 °C until further use.

METHOD DETAILS

SARS-CoV-2 pseudovirus neutralization assay

The pseudovirus neutralization assay was performed as described previously(Caniels et al., 2021). Shortly, HEK293T/ACE2 cells were kindly provided by P. Bieniasz(Schmidt et al., 2020) were seeded at a density of 20,000 cells per well in a 96-well plate coated with poly-lysine (50 ug/ml) 1 day before the start of the neutralization assay. The next day, heat-inactivated sera samples were in triplicate serial diluted in threefold steps, starting at 1:20 dilution to test for Omicron BA.1 and BA.2 pseudovirus neutralization and 1:100 for all the other variants. Sera was diluted in cell culture medium (DMEM), supplemented with 10% fetal bovine serum, penicillin (100 U/ml), streptomycin (100 g/ml), and GlutaMAX (Gibco), mixed in a 1:1 ratio with pseudovirus, and incubated for 1 hour at 37°C. These mixtures were then added to the cells in a 1:1 ratio and incubated for 48 hours at 37°C, and lysis buffer was added. The luciferase activity in cell lysates was measured using the Nano-Glo Luciferase Assay System (Promega) and GloMax system (Turner BioSystems). Relative luminescence units were normalized to those from cells infected with SARS-CoV-2 pseudovirus in the absence of sera. The inhibitory neutralization titres (ID_{50}) were determined as the serum dilution at which infectivity was inhibited by 50%, using a nonlinear regression curve fit (GraphPad Prism software version 8.3). The International Standard for anti-SARS-CoV-2 immunoglobulins provided by the WHO(Kristiansen et al., 2021) were used to convert the ID_{50} values into International Units per milliliters (IU/mL). Samples with IU/mL titres <10 were defined as having undetectable neutralization against the D614G, Alpha, Beta, Gamma and Delta variant. For Omicron BA.1 and BA.2 neutralization, the start-dilution of 1:20 enables a cut-off of <2 IU/mL for all samples except for some Alpha infected individuals. A limited amount of sera was available from the Alpha infected individuals, resulting in a start dilution of 1:100 of n=7 samples against all variants including Omicron BA.1 and BA.2. Neutralization data points of two Alpha infected individuals against BA.1 and BA.2 were excluded from Figure 1A because a neutralization titres <10IU/mL (Table S2). This exclusion did not impact the statistics as written below because a general cut-off of <10IU/mL were used for neutralization against any variant, including Omicron BA.1 and BA.2.

Antigenic cartography

Antigenic maps were constructed as previously described (Fonville et al., 2014; Smith et al., 2004) using the antigenic cartography software from https://acmacs-web.antigenic-cartography.org. In brief, this approach to antigenic mapping uses multidimensional scaling to position antigens (viruses) and sera in a map to represent their antigenic relationships. The maps here relied on the SARS-CoV-2 post-infection serology data and post-vaccination serology data shown in Figure 1A and Table S2. The positions of antigens and sera were optimized in the map to minimise the error between the target distances set by the observed pairwise virus-serum combinations in the pseudovirus assay described above and the resulting computationally derived map. Maps were constructed in 2, 3, 4, and 5 dimensions to investigate the dimensionality of the antigenic relationships. Both the convalescent (Figure 3A)

CellPress
OPEN ACCESS

and post-vaccination datasets (Figure 3B) were strongly two-dimensional with only small improvements in residual mean squared error of the maps as map dimensionality increased.

QUANTIFICATION AND STATISTICAL ANALYSIS

Data visualization and statistical analyses were performed in GraphPad Prism software (version 8.3). Spider plots (Figure 1B) were made in Excel 2016. The antigenic maps were produced using the antigenic cartography software mentioned above. Wilcoxon signed rank test with Benjamini Hochberg correction was used to compare cross-neutralization titres with the homologous neutralization (Figure 1A). Mann-Whitney test was used for non-paired group comparisons (Figure 2). All statistics mentioned here were performed by using a general neutralization cut-off of 10IU/mL against any variant of SARS-CoV-2.

Immunity, Volume *55*

Supplemental information

Antigenic cartography using sera from sequence-confirmed SARS-CoV-2 variants of concern infections reveals antigenic divergence of Omicron

Karlijn van der Straten, Denise Guerra, Marit J. van Gils, Ilja Bontjer, Tom G. Caniels, Hugo D.G. van Willigen, Elke Wynberg, Meliawati Poniman, Judith A. Burger, Joey H. Bouhuijs, Jacqueline van Rijswijk, Wouter Olijhoek, Marinus H. Liesdek, A.H. Ayesha Lavell, Brent Appelman, Jonne J. Sikkens, Marije K. Bomers, Alvin X. Han, Brooke E. Nichols, Maria Prins, Harry Vennema, Chantal Reusken, Menno D. de Jong, Godelieve J. de Bree, Colin A. Russell, Dirk Eggink, and Rogier W. Sanders

This PDF file includes:

Supplementary Figure 1 (Figure S1)
Supplementary Figure 2 (Figure S2)
Supplementary Table 2 (Table S2)
Supplementary Table 3 (Table S3)

Included as a separate excel file:

Supplementary Table 1 and corresponding legend (Table S1)

Supplementary Figure 1. Antigenic cartography reveals antigenic diversification of SARS-CoV-2, related to Figure 3A

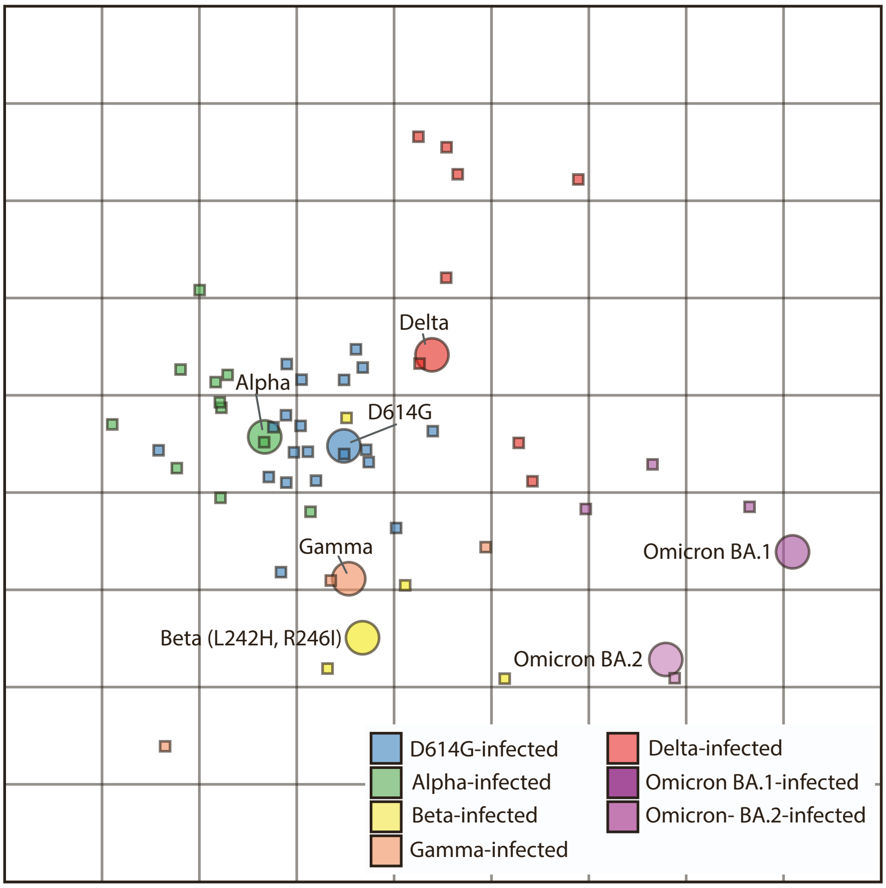

Antigenic map of SARS-CoV-2 VOCs based on convalescent SARS-CoV-2 infection sera. SARS-CoV-2 variants are shown as circles and sera are indicated as squares. Each square corresponds to sera of one individual and is coloured by the infecting SARS-CoV-2 variant. Both axes of the map are antigenic distance and each grid square (1 antigenic unit) represents a two-fold change in neutralization titre. The distance between points in the map can be interpreted as a measure of antigenic similarity, where the points more closely together show higher cross-neutralization and are therefore antigenically more similar. Compared to Figure 3A, this panel does not contain the Beta (Δ242-244) subvariant.

Supplementary Figure 2. Neutralization of convalescent VOC sera against two Beta subvariant pseudoviruses, related to Figure 1A

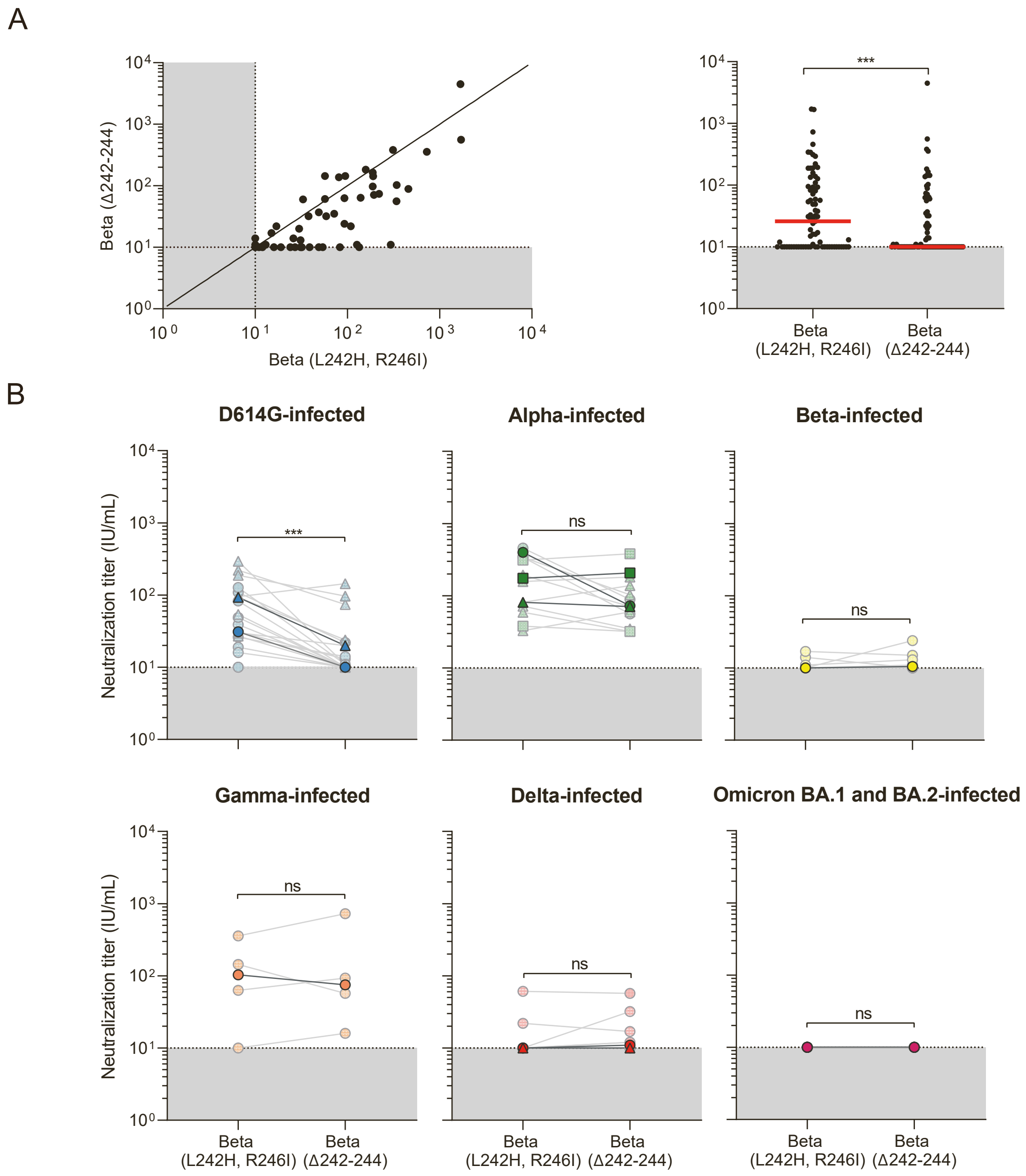

A. The left panel shows the correlation between the midpoint neutralization titres against both Beta subvariants used in this research, expressed in International Units per mL (IU/mL). In the right panel we studied the difference in neutralization titre against both Beta subvariants using a Wilcoxon signed rank test. Median neutralization titres are depicted as red bars. *** = p<0.001. B. Midpoint neutralization titres against both Beta subvariants in international Units per mL (IU/mL). The individuals are grouped per VOC they were infected with and plotted accordingly. Non-hospitalized patients are indicated with dots and hospitalized patients with triangles. The individuals that were infected with an Alpha variant that also included the E484K mutation are indicated in green squares. Median neutralization titres were compared using a Wilcoxon signed rank test. ns= non-significant, *** = p< 0.001.

Supplementary Table 2. Neutralization titres of convalescent SARS-CoV-2 sera against several VOCs, related to Figure 1-3.

Neutralization titres (ID50) were the serum dilution at which infectivity was inhibited 50% are converted to International Units per millilitre (IU/mL). Neutralization titers of post-vaccination sera can be found in Van Gils et al, Plos Medicine, 2022.

Infected strain	Participant ID	D614G	Alpha	Beta (L242H, R246I)	Beta (Δ242-244)	Gamma	Delta	Omicron BA.1	Omicron BA.2
D614G	COSCA-020	233	37	109	22	72	54	<2	81
D614G	COSCA-021	231	58	39	<10	35	29	<2	6
D614G	COSCA-022	28	11	<10	<10	<10	23	<2	<2
D614G	COSCA-023	34	13	19	<10	15	28	<2	3
D614G	COSCA-024	90	106	31	13	61	61	<2	7
D614G	COSCA-025	69	47	49	10	77	122	<2	9
D614G	COSCA-026	101	83	83	<10	38	97	<2	4
D614G	COSCA-028	189	119	26	14	81	217	21	55
D614G	COSCA-033	48	29	16	<10	28	36	<2	4
D614G	COSCA-034	34	27	<10	<10	<10	<10	<2	4
D614G	COSCA-112	929	1133	221	74	386	890	7	67
D614G	COSCA-113	122	97	31	<10	89	134	3	24
D614G	COSCA-114	237	249	127	11	131	68	3	15
D614G	COSCA-115	601	1095	93	24	386	384	112	13
D614G	COSCA-116	510	1005	188	97	482	230	9	157
D614G	COSCA-117	223	206	27	<10	92	48	5	7
D614G	COSCA-118	432	438	95	144	221	814	41	13
D614G	COSCA-119	430	220	30	20	186	326	11	19
D614G	COSCA-120	335	234	295	11	372	134	64	59
D614G	COSCA-123	852	463	54	<10	393	586	8	18
Alpha	AMCVIS1585	607	1366	194	71	436	344	23	13
Alpha	AMCVIS1871	215	683	342	103	414	557	20	<10
Alpha	AMCVIS2084	291	891	158	182	171	482	<10	11

Alpha	AMCVIS3484	124	872	33	60	93	232	13	<10
Alpha	AMCVIS5722	164	394	72	35	45	137	<10	<10
Alpha	AMCVIS9584	168	733	59	32	103	55	54	23
Alpha	COSCA-303	229	2103	341	56	122	108	40	16
Alpha	GGDVIS3343	1083	1658	458	89	1138	159	148	92
Alpha	VUVIS6992	263	788	81	138	149	252	4	<10
Alpha + E484K	COSCA-316	558	2091	312	382	730	181	24	36
Alpha + E484K	COSCA-320	59	213	38	32	82	16	9	6
Beta	COSCA-331	<10	<10	<10	14	<10	<10	<2	<2
Beta	COSCA-336	<10	16	15	17	34	<10	5	3
Beta	COSCA-337	<10	<10	<10	<10	<10	<10	2	5
Gamma	COSCA-309	57	51	57	144	87	<10	4	10
Gamma	COSCA-310	<10	28	93	63	224	<10	<2	4
Gamma	COSCA-324	<10	<10	16	<10	22	<10	<2	<2
Gamma	COSCA-334	537	326	726	357	761	403	798	725
Delta	COSCA-321	<10	14	<10	<10	<10	217	10	8
Delta	COSCA-322	11	10	<10	<10	<10	159	3	2
Delta	COSCA-323	50	54	12	<10	16	110	5	12
Delta	COSCA-325	105	37	<10	<10	<10	439	5	9
Delta	COSCA-327	<10	<10	<10	<10	<10	<10	<2	<2
Delta	COSCA-328	72	38	32	<10	21	150	70	30
Delta	COSCA-329	<10	<10	<10	<10	<10	19	<2	<2
Delta	COSCA-330	<10	<10	17	22	12	<10	<2	<2
Delta	COSCA-332	10	<10	<10	<10	10	59	<2	4
Delta	COSCA-333	114	78	57	61	121	1635	32	27
Delta	COSCA-335	<10	<10	12	<10	<10	27	8	7
Beta	COSCA-301	17	12	<10	<10	<10	11	<2	<2
Beta	COSCA-305	<10	<10	24	<10	67	<10	8	36

Beta	COSCA-306	<10	<10	11	<10	17	<10	<2	<2
Beta	COSCA-307	<10	<10	13	11	32	<10	2	2
Beta	COSCA-308	<10	<10	<10	<10	<10	<10	<2	<2
Omicron BA.1	COSCA-348	<10	<10	<10	<10	<10	<10	5	<2
Omicron BA.1	COSCA-349	<10	<10	<10	<10	<10	<10	24	15
Omicron BA.1	COSCA-350	<10	<10	<10	<10	<10	<10	61	21
Omicron BA.1	COSCA-351	<10	<10	<10	<10	<10	<10	22	2
Omicron BA.1 / BA.2	COSCA-352	<10	<10	<10	<10	<10	12	23	5
Omicron BA.1 / BA.2	COSCA-353	<10	<10	<10	<10	<10	11	90	22
Omicron BA.1	COSCA-354	<10	<10	<10	<10	<10	10	4	9
Omicron BA.2	COSCA-356	<10	16	<10	<10	<10	<10	<2	32
Omicron BA.2	COSCA-357	<10	<10	<10	<10	<10	<10	15	95
Omicron BA.2	COSCA-358	14	<10	<10	<10	<10	<10	16	95
Omicron BA.1	COSCA-360	<10	<10	<10	<10	<10	<10	3	37
Omicron BA.2	COSCA-361	<10	<10	<10	<10	<10	<10	<2	7

Supplementary Table 3. Exact p-values of statistical analysis of Figure 1A, Figure 2A and Figure S1.

Supplementary Table 3A. Statistical analysis of Figure 1A.

	**Infected individuals**						
	**Ancestral**	**Alpha**	**Beta**	**Gamma**	**Delta**	**Omicron BA.1**	**Omicron BA.2**
**Ancestral**		0,0010	0,6000	0,1750	0,0046	0,0729	0,25
**Alpha**	0,1208		0,6000	0,1750	0,0046	0,0729	0,25
**Beta (L242H, R246I)**	0,0002	0,0010		0,1750	0,0046	0,0729	0,25
**Beta (Δ242-244)**	0,0002	0,0010		0,3750	0,0059	0,0729	0,25
**Gamma**	0,0002	0,0010	0,3750		0,0046	0,0729	0,25
**Delta**	0,0183	0,0010	0,3750	0,1750		0,0729	0,25
**Omicron BA.1**	0,0001	0,0010	0,3750	0,3750	0,0046		0,25
**Omicron BA.2**	0,0001	0,0010	0,8750	0,1750	0,0046	0,2188	

ns	P > 0.05
*	P ≤ 0.05
**	P ≤ 0.01
***	P ≤ 0.001

Supplementary Table 3B. Statistical analysis of Figure 2A.

**P-values using Mann-Whitney U test**	**P-value**	
**Hospitalized patients**		
D614G vs Alpha infected	0,11	ns
**Non-hospitalized**		
D614G vs Beta(Δ242-244) infected	<0,0001	****
D614G vs Delta infected	0,46	ns
Beta (Δ242-244) vs Omicron BA.1 infected	0,088	ns

Supplementary Table 3C. Statistical analysis of Figure S1.

**P-values using Wilcoxon rank test**							
	Ancestral	Alpha	Beta	Gamma	Delta	Omicron BA.1	Omicron BA.2
Beta (L242H, R246I) vs Beta (Δ242-244)	0,0004	0,21	0,875	0,625	>0,99	>0,99	>0,99
